# Metabolic interplay between exogenous cystine and glutamine dependence in triple-negative breast cancer

**DOI:** 10.1038/s41420-025-02714-3

**Published:** 2025-10-06

**Authors:** Ziqian Ge, Martina Wallace, Rory Turner, Maureen Yin, Mary F. Rooney, Richard K. Porter

**Affiliations:** 1 School of Biochemistry and Immunology, Trinity Biomedical Sciences Institute, Trinity College Dublin, Dublin, Ireland; 2 School of Agriculture and Food Science, University College Dublin, Belfield, Dublin, Ireland; 3 Conway Institute of Biomolecular and Biomedical Research, University College Dublin, Dublin, Ireland; 4 Department of Cell and Molecular Biology, Karolinska Institute, Stockholm, Sweden

**Keywords:** Cancer metabolism, Cell death

## Abstract

Triple-negative breast cancer (TNBC) is an aggressive breast cancer subtype characterized by high recurrence rates and limited treatment options due to the absence of hormone receptors. Despite advancements in breast cancer research, effective therapies for TNBC remain inadequate, highlighting the need to elucidate subtype-specific metabolic vulnerabilities. TNBC cells exhibit a strong dependence on the exogenous amino acids cystine and glutamine, yet the interplay between these metabolic dependencies remains poorly understood. Here, we demonstrate that TNBC cells exhibit sensitivity to individual nutrient deprivation but can survive dual cystine and glutamine deprivation via distinct mechanisms. Exogenous glutamine primarily fuels glutamine anaplerosis, supporting TNBC cell proliferation. Notably, when exogenous glutamine is absent, restricted cystine uptake restores intracellular glutamate levels, fulfilling metabolic demands and sustaining TNBC cell growth. Under cystine deprivation, inhibition of glutaminolysis rescues TNBC cells by mitigating lipid peroxidation and reducing ROS production, whereas supplementation with the TCA cycle intermediates ɑ-ketoglutarate (ɑ-KG) and succinate induces profound cell death in both TNBC and luminal breast cancer cells under glutaminolysis blockade. Collectively, these findings highlight the metabolic interdependence of glutamine and cystine in TNBC, providing mechanistic insights into potential metabolic-targeted and dietary interventions for TNBC therapy.

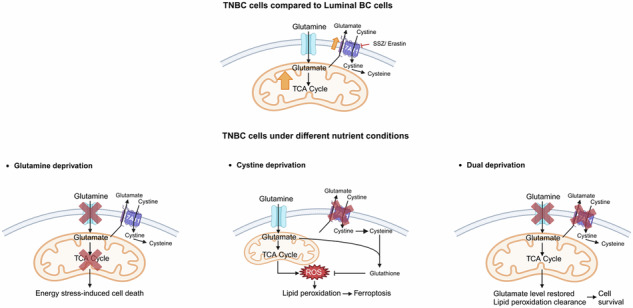

## Introduction

Triple-negative breast cancer (TNBC) accounts for ~10–20% of all breast cancer cases and is characterized by aggressive growth, high recurrence rates, and limited treatment options due to the absence of estrogen receptor (ER), progesterone receptor (PR), and human epidermal growth factor receptor 2 (HER2) [1, 2]. Despite progress in breast cancer research, effective therapies for TNBC remain inadequate, highlighting the urgent need for novel treatment strategies. One promising avenue involves targeting TNBC’s altered metabolic landscape, which differs markedly from that of luminal breast cancer, a subtype defined by the presence of hormone receptors (ER and/or PR) and useally linked to a less aggressive phenotype [3, 4]. TNBC cells are known to exhibit a distinct metabolic profile, particularly a strong dependency on glutamine and cystine [5–7].

The interplay between glutamine metabolism and cystine availability, particularly through the activity of the cystine/glutamate antiporter SLC7A11, has been highlighted in several studies. Some studies have shown that either genetic inactivation of SLC7A11 or reduction of cystine availability in the culture medium can rescue cells from nutrient stress. This rescue occurs through restoration of intracellular glutamate levels, which are redirected toward the tricarboxylic acid (TCA) cycle to support energy production under conditions of glucose or glutamine restriction [8–10]. While the inactivation of SLC7A11 exhibited a rescue effect under nutrient limitation, the essential role of glutamate and cystine for glutathione synthesis highlighted the anti-tumor effect of inhibition of SLC7A11. Timmerman et al. [7] demonstrated the anti-tumor effect in TNBC cells of SLC7A11 inhibitor sulfasalazine (SSZ) through interrupting redox homeostasis in glutamine-addicted TNBC cells. Additionally, dual metabolic inhibition of glutaminase (GLS) and SLC7A11 induces apoptosis and ferroptosis in TNBC cells [11]. On the other hand, glutamine anaplerosis was recognized as an essential factor for cystine deprivation-induced ferroptosis [12, 13].

While the importance of exogenous glutamine and cystine in TNBC metabolism is well established, their functional interplay in relation to glutamine anaplerosis remains underexplored. Using the highly invasive Hs578Ts(i)_8_ subclone [14], we showed that this TNBC line depends on glutamine-driven TCA cycle activity for survival. Remarkably, inactivation of SLC7A11 or cystine deprivation rescues cell viability by preserving intracellular glutamate. A similar rescue effect was observed in another glutamine-dependent TNBC line, SUM159. Conversely, pharmacological inhibition of glutamine anaplerosis under cystine-deprived conditions reduces ROS and lipid peroxidation, providing an alternative rescue mechanism. Notably, supplementation with α-KG or succinate under dual nutrient restriction induces cell death in both TNBC and luminal breast cancer cells, suggesting subtype-specific sensitivity linked to glutaminolysis-driven TCA cycle activity. Overall, our findings highlight glutamine and cystine co-dependence as a metabolic vulnerability in TNBC and reveal adaptive responses that may be therapeutically targeted.

## Results

### Elevated dependence on extracellular glutamine and cystine in TNBC cells

**Fig. 1:**
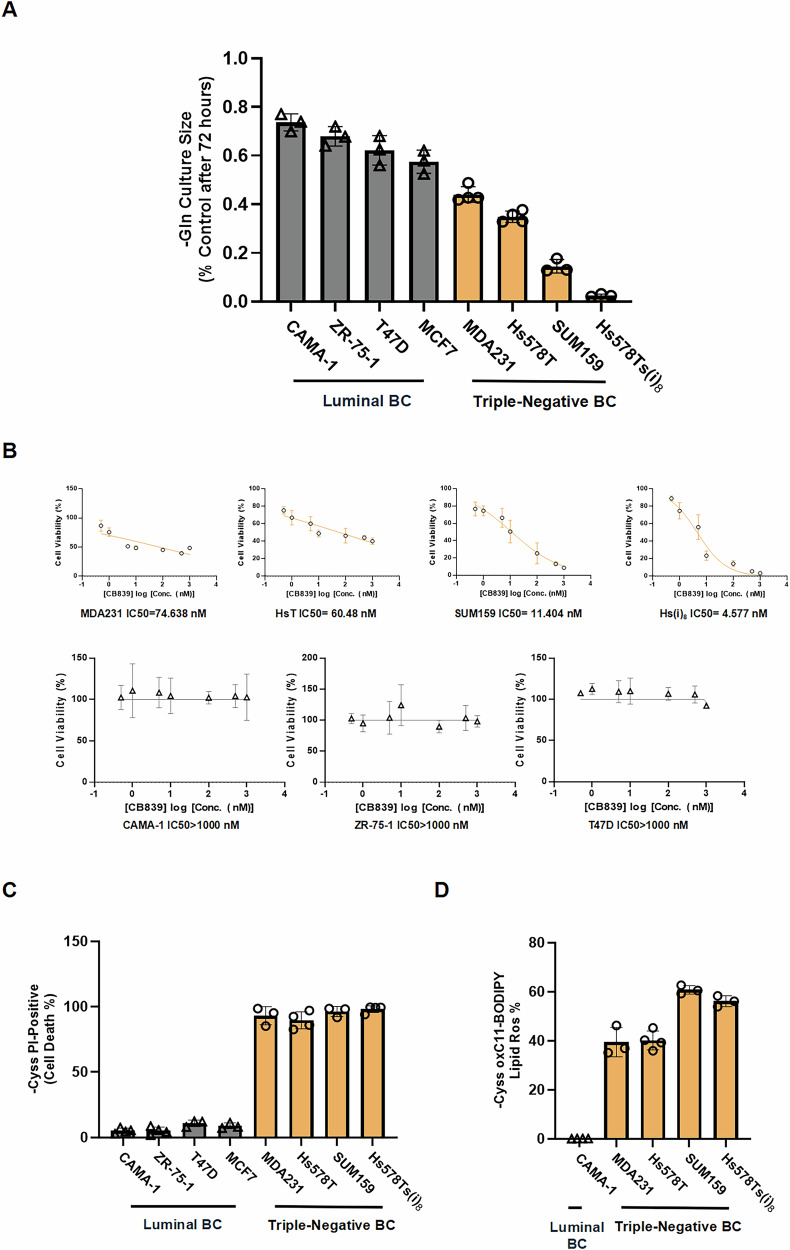
Elevated dependence on extracellular glutamine and cystine in TNBC cells. **A** Relative proliferation of indicated luminal and TNBC cell lines cultured in glutamine-deprived medium for 72 h, normalized to control conditions (*n* = 3–4). **B** Sensitivity of indicated cell lines to the glutaminase inhibitor CB839 (*n* = 3). **C** Cystine deprivation-induced cell death across cell lines after 48 h, assessed by the proportion of PI-positive cells. All PI staining related to cystine deprivation, including combination treatments, was measured at 48 h (*n* = 3–4). **D** Lipid peroxidation following 16 h of cystine deprivation, measured by the percentage of oxidized BODIPY-C11-positive cells. All lipid peroxidation measurements were performed after 16 h of treatment (*n* = 3–4). Data represent mean ± SD.

To investigate the dependence of breast cancer (BC) cells on glutamine (Gln), we first assessed cell proliferation under Gln deprivation and the sensitivity to the glutaminase (GLS1) inhibitor CB839 in luminal BC cell lines (CAMA-1, ZR-75-1, T47D, and MCF7) and TNBC cell lines (MDA-MB-231, SUM159, Hs578T, and its invasive subclone Hs578Ts(i)_8_) after 72 h of culture (Fig. 1A, B). TNBC cells exhibited a greater reliance on exogenous Gln and higher sensitivity to CB839 compared to luminal BC cells. Among all TNBC lines, Hs578Ts(i)_8_ cells displayed the highest dependency on glutamine for proliferation and the greatest sensitivity to CB839. Hs578Ts(i)_8_ is an invasive subclone of Hs578T, selected through eight rounds of transwell invasion assay, and exhibits greater migratory and invasive potential than its parental Hs578T cells (Fig. S1.1A–C). Furthermore, CB839 treatment significantly inhibited cell invasion in both Hs578T and Hs578Ts(i)_8_ cells, with a more pronounced effect in Hs578Ts(i)_8_ cells. This finding suggests that extracellular glutamine plays a critical role not only in TNBC cell proliferation but also in invasion potential (Fig. S1.1D). Additionally, public datasets from CCLE and HMS LINCS revealed elevated GLS1 RNA expression in TNBC cells, a finding further validated via western blot analysis of the cell lines used in this study (Fig. S1.2A–C, original blots shown in Fig. S5A). Next, we examined the dependency of TNBC cells on cystine (Cyss), another amino acid critical for TNBC survival. Cyss deprivation for 48 h resulted in rapid cell death in TNBC but not luminal BC cells (Fig. 1C). This Cyss deprivation-induced TNBC cell death, characteristic of ferroptosis, was associated with lipid peroxidation accumulation (Fig. 1D) and was rescued by the ferroptosis inhibitor Fer-1 (Fig. S1.2E).

### Enhanced glutamine anaplerosis underlies extracellular glutamine dependency in TNBC cells

**Fig. 2:**
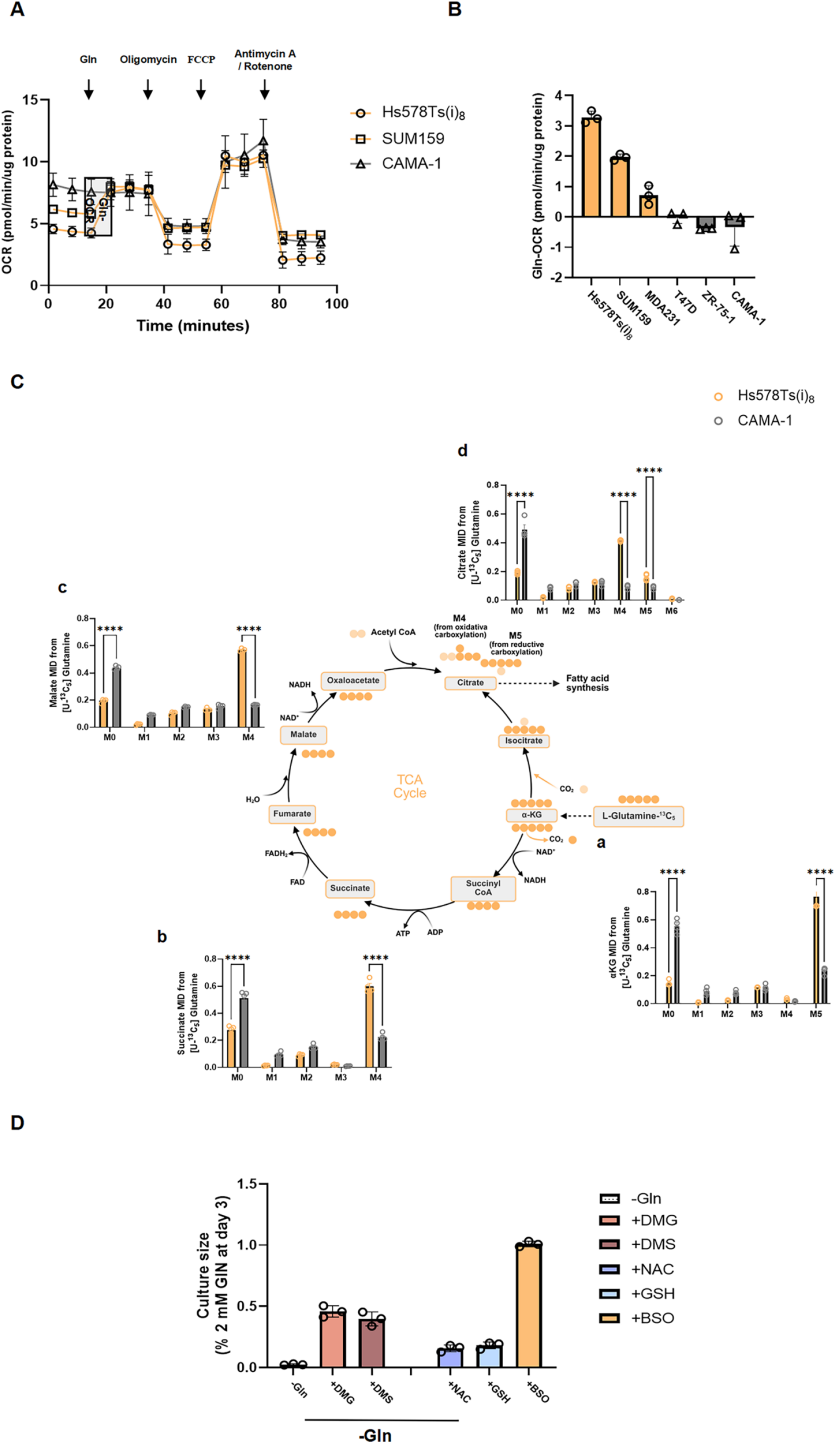
Enhanced glutamine anaplerosis underlies extracellular glutamine dependency in TNBC cells. **A** Real-time OCR following sequential injections of glutamine (2 mM), oligomycin (1.5 µM), FCCP (2 µM for Hs578Ts(i)_8_ and SUM159 cells; 0.5 µM for CAMA-1), and a combination of antimycin A (0.5 µM) and rotenone (0.5 µM). OCR is normalized to protein content and expressed as pmol/min/µg protein. **B** Change in OCR after glutamine injection (Gln-OCR) across luminal and TNBC cell lines; Gln-OCR is calculated by subtracting the average pre-injection OCR from the average post-injection OCR (*n* = 3). **C** Contribution of extracellular glutamine to TCA cycle intermediates in Hs578Ts(i)_8_ and CAMA-1 cells, represented as isotopologue distribution (M0–M5) of (a) α-KG, (b) succinate, (c) malate, and (d) citrate from U-^13^C_5_-glutamine tracing (*n* = 4–5). **D** Effects of TCA cycle intermediates (DMG: 6 mM; DMS: 12 mM) and antioxidants (NAC: 2 mM; GSH: 2 mM) on proliferation under glutamine-deprived conditions, and the effect of glutathione synthesis inhibition (BSO: 50 µM) on proliferation in Hs578Ts(i)_8_ cells after 72 h (*n* = 3). Data represent mean ± SD. Statistical analysis in (**C**) was performed using two-way ANOVA followed by Šídák’s multiple comparisons test; ****p < 0.0001.

To elucidate the mechanism underlying glutamine dependency in TNBC cells, we first assessed oxygen consumption rate (OCR) in TNBC and luminal BC cells, given that glutamine serves as a critical carbon source for replenishing the tricarboxylic acid (TCA) cycle. Using the Agilent Seahorse XF Analyzer, we measured the real-time oxygen consumption in response to glutamine supplementation in luminal BC cell lines (CAMA-1, ZR-75-1, and T47D) and TNBC cell lines (MDA-MB-231, SUM159, Hs578T, and its invasive subclone Hs578Ts(i)_8_) (Figs. 2A and S2A, B). Gln-OCR was calculated by subtracting the baseline OCR from the OCR post-glutamine injection. Notably, a significant increase in OCR following glutamine injection was observed exclusively in TNBC cells, with Hs578Ts(i)_8_ exhibiting the most pronounced change (Fig. 2B). To further investigate glutamine metabolism in TNBC, we performed stable isotope-labelled glutamine (U-^13^C_5_-glutamine) tracing using GC-MS in the most glutamine-dependent TNBC cell line (Hs578Ts(i)_8_) and the least glutamine-dependent luminal BC cell line (CAMA-1). Compared to CAMA-1, Hs578Ts(i)_8_ incorporated less glutamine-derived carbon into the TCA cycle (Fig. 2C). Notably, Hs578Ts(i)_8_ exhibited increased M + 4 and M + 5 in citrate, indicating the cell may be utilising glutamine for both oxidative and reductive carboxylation pathways, suggesting elevated utilization of glutamine for lipid synthesis [15]. Given that glutamine also contributes to antioxidant defence via glutathione (GSH) synthesis, we tested whether glutamine deprivation-induced proliferation defects were due to GSH depletion by supplementing Hs578Ts(i)_8_ cells with exogenous GSH or N-acetylcysteine (NAC). However, neither antioxidant rescued proliferation under glutamine deprivation. In contrast, supplementation with cell-permeable TCA cycle intermediates dimethyl α-ketoglutarate (DMG) and dimethyl succinate (DMS) effectively restored proliferation under glutamine-deprived conditions. Consistently, treatment with the glutathione synthesis inhibitor buthionine sulfoximine (BSO) did not affect proliferation under basal conditions (Fig. 2D), further supporting that glutamine reliance in TNBC is primarily driven by anaplerosis rather than antioxidant requirements. Taken together, our results demonstrate that TNBC cells exhibit a heightened demand for glutamine anaplerosis under basal conditions, while glutamine supplementation may become essential for survival under oxidative stress.

### Cystine restriction rescues glutamine-addicted TNBC cells from glutamine deprivation by restoring intracellular TCA cycle intermediates

**Fig. 3:**
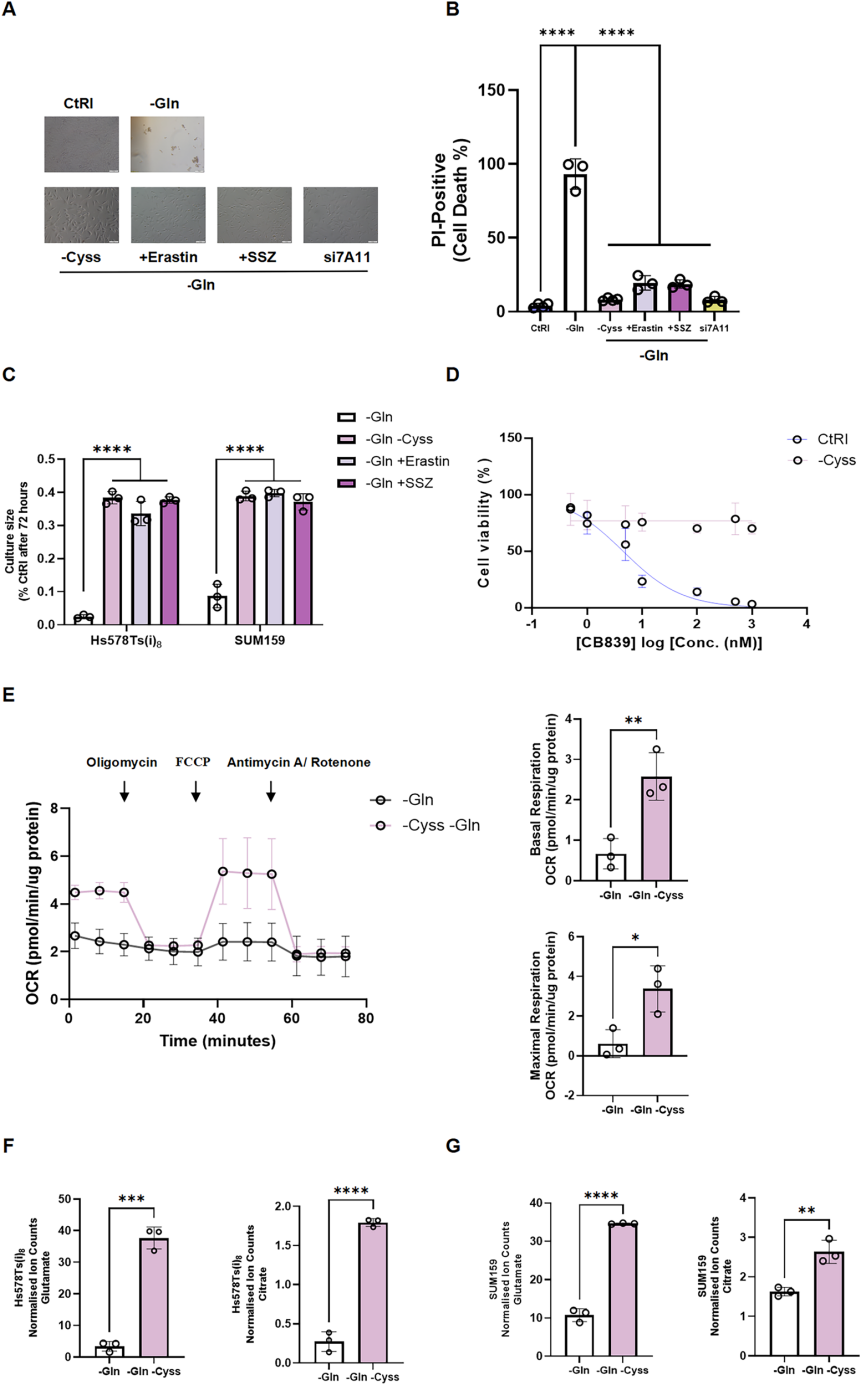
Cystine restriction rescues glutamine-addicted TNBC cells from glutamine deprivation by restoring intracellular TCA cycle intermediates. **A** Representative phase-contrast images of TNBC cells under glutamine-deprived conditions with concurrent cystine deprivation or SLC7A11 inactivation via erastin (1 µM), sulfasalazine (50 µM), or SLC7A11 knockdown (scale bar = 100 µm). **B** Quantification of cell death by PI-positive cells under the same treatment conditions as in (**A**). **C** Rescue of cell proliferation by pharmacological SLC7A11 inhibition or cystine deprivation in SUM159 and Hs578Ts(i)_8_ cells after 72 h. **D** Effect of cystine deprivation on the sensitivity of Hs578Ts(i)_8_ cells to CB839 over 72 h. **E** Real-time OCR measurement following 8-h pretreatment in glutamine-deprived or glutamine- and cystine-deprived media, with sequential injections of oligomycin (1.5 µM), FCCP (2 µM), and antimycin A/rotenone (0.5 µM each) in Hs578Ts(i)_8_ cells. OCR was normalized to protein content and expressed as pmol/min/µg protein. Basal respiration (OCR before injections minus non-mitochondrial respiration) and maximal respiration were calculated and presented as bar graphs. Normalized intracellular levels of glutamate and citrate under glutamine deprivation or dual glutamine and cystine deprivation in **F** Hs578Ts(i)_8_ and **G** SUM159 cells. Data represent mean ± SD (*n* = 3). Statistical analyses were performed using one-way ANOVA with Tukey’s multiple comparisons test (**B**), two-way ANOVA with Šídák’s multiple comparisons test (**C**), or unpaired *t*-test (bar charts in **E**–**G**). ****p < 0.0001, **p < 0.01, *p < 0.05.

Given that TNBC cells exhibit heightened reliance on extracellular glutamine due to enhanced glutamine anaplerosis, we next investigated whether this phenotype is linked to an increased dependence on extracellular cystine. Analysis of cystine/glutamate transporter expression in BC cells using public databases CCLE and HMS LINCS revealed that SLC7A11 is overexpressed in TNBC cells, with Hs578T and SUM159 being among the highest-expressing cell lines in the TNBC cohort (Fig. S3A, B). This finding was further validated by western blot analysis (Fig. S3C, original western blots shown in Fig. S5B). To assess the role of SLC7A11 and cystine availability under glutamine deprivation, we focused on the most glutamine-anaplerosis-dependent TNBC cell line, Hs578Ts(i)_8_. Upon glutamine deprivation, which induces severe cell death, we found that SLC7A11 inactivation, either by gene knockdown (efficiency shown in Fig. S3D, original western blots shown in Fig. S5B) or pharmacological inhibition, as well as cystine deprivation, rescued Hs578Ts(i)_8_ cells under these conditions (Fig. 3A, B). Similarly, intracellular cystine restriction also rescued cell proliferation in SUM159 cells under glutamine deprivation (Fig. 3C). Furthermore, cystine deprivation in the culture medium reduced the sensitivity of Hs578Ts(i)_8_ cells to the CB839 glutaminase inhibitor (Fig. 3D), highlighting the interplay between cystine and glutamine metabolism in TNBC cells. Since SLC7A11 exports glutamate, and considering the increased demand for glutamate in the TCA cycle, we hypothesized that restriction of intracellular cystine (via SLC7A11 inactivation or extracellular cystine depletion) restores intracellular glutamate levels, thereby maintaining energy homeostasis for TNBC cell survival under glutamine-deprived conditions. To test this hypothesis, we conducted Seahorse assays to measure OCR and GC–MS to assess intracellular TCA cycle intermediates. We observed increased basal and maximal OCR (Fig. 3E) and elevated levels of TCA cycle intermediates after 6 h treatment (Fig. 3F, G) under combined cystine and glutamine deprivation compared to glutamine deprivation alone in TNBC cells, whereas these metabolic changes were not observed in CAMA-1 cells (Fig. S3E).

### Glutamine availability is crucial for cystine deprivation-induced cell death

**Fig. 4:**
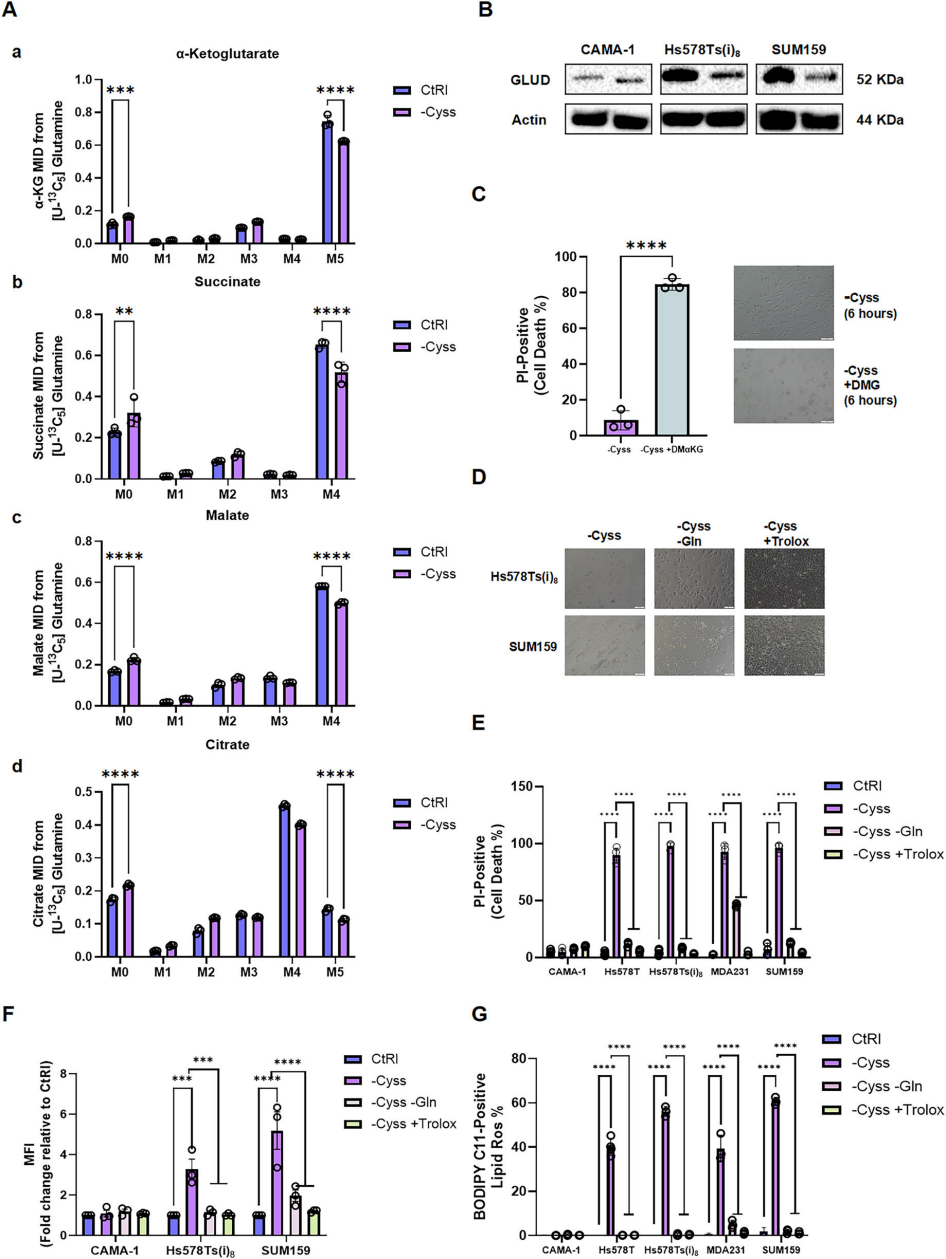
Glutamine availability is crucial for cystine deprivation-induced cell death. **A** Contribution of extracellular glutamine to TCA cycle intermediates (a) α-KG, (b) succinate, (c) malate, and (d) citrate under control and cystine-deprived conditions in Hs578Ts(i)_8_ cells, assessed by U-^13^C_5_-glutamine tracing (*n* = 3). **B** Western blot analysis of GLUD under the indicated conditions. Original blots were shown in Fig. S5C. **C** Effect of DMG (6 mM) on cell viability after 6 h under cystine-deprived conditions, assessed by PI-positive cell percentage and representative phase-contrast images (scale bar = 100 µm). **D** Representative images of Hs578Ts(i)_8_ and SUM159 cells treated with Trolox (5 µM) and/or glutamine deprivation under cystine-deprived conditions. Corresponding quantification of **E** cell death (PI-positive cells), **F** cellular ROS levels (median fluorescence intensity of DCF, normalized to control), and **G** lipid peroxidation (percentage of oxidized BODIPY-C11-positive cells) (*n* = 3–5). Data represent mean ± SD. Statistical analysis was performed using two-way ANOVA followed by Šídák’s multiple comparisons test (**A**, **E–G**), or unpaired *t*-test (bar graph in **C**). ****p < 0.0001, ***p < 0.001.

Next, we investigated the effect of cystine deprivation on glutaminolysis. A reduction in GLS1 RNA expression upon cystine deprivation in Hs578T cells was previously reported by Bottoni et al. [5]. To assess the direct effect of cystine deprivation on glutamine anaplerosis, we performed U-^13^C_5_-glutamine isotope tracing using GC–MS and observed a reduction in glutamine anaplerosis after 6 h of cystine deprivation in Hs578Ts(i)_8_ cells (Fig. 4A). To further support this observation, we detected a decreased protein level of GLUD (Fig. 4B), a key enzyme in glutamine anaplerosis that catalyzes the conversion of glutamate to α-ketoglutarate. These findings suggest that while cystine deprivation restores TCA cycle activity under glutamine deprivation, it inhibits glutamine anaplerosis on its own. We then assessed whether cystine deprivation-induced cell death in TNBC cells could be partially attributed to the decreased glutamine anaplerosis. To test this, we added DMG, a cell-permeable α-KG, which had previously rescued cells under glutamine deprivation, to Hs578Ts(i)_8_ cells under cystine deprivation to restore TCA cycle activity. Surprisingly, DMG addition under cystine deprivation resulted in significantly more cell death after 6 h compared to cystine deprivation alone (Fig. 4C). Since mitochondrial respiration is a critical source of ROS, we hypothesized that DMG addition disrupted the balance between ROS production and antioxidant capacity under cystine deprivation. Indeed, decreased ROS levels were observed under cystine and glutamine deprivation compared to cystine deprivation alone. Antioxidant treatment with Trolox also rescued TNBC cells from cystine deprivation-induced cell death by reducing ROS levels (Fig. 4D–F). A decrease in lipid peroxidation levels was observed under combined cystine and glutamine deprivation compared to cystine deprivation alone (Fig. 4G). Furthermore, treatment with the mitophagy inducer FCCP rescued Hs578Ts(i)_8_ cells from cystine deprivation-induced cell death, accompanied by a reduction in lipid peroxidation. Both FCCP treatment and glutamine deprivation under cystine-deprived conditions induced expression of mitophagy markers, including PINK1, and were associated with a reduction in mitochondrial marker TOMM20 (Fig. S4A–D, original blots shown in Fig. S5D, E). Taken together, these findings suggest that glutamine-related mitochondrial activity plays a critical role in regulating cystine deprivation-induced cell death in TNBC cells.

### Glutamine anaplerosis regulates cystine deprivation-induced cell death by modulating lipid peroxidation

**Fig. 5:**
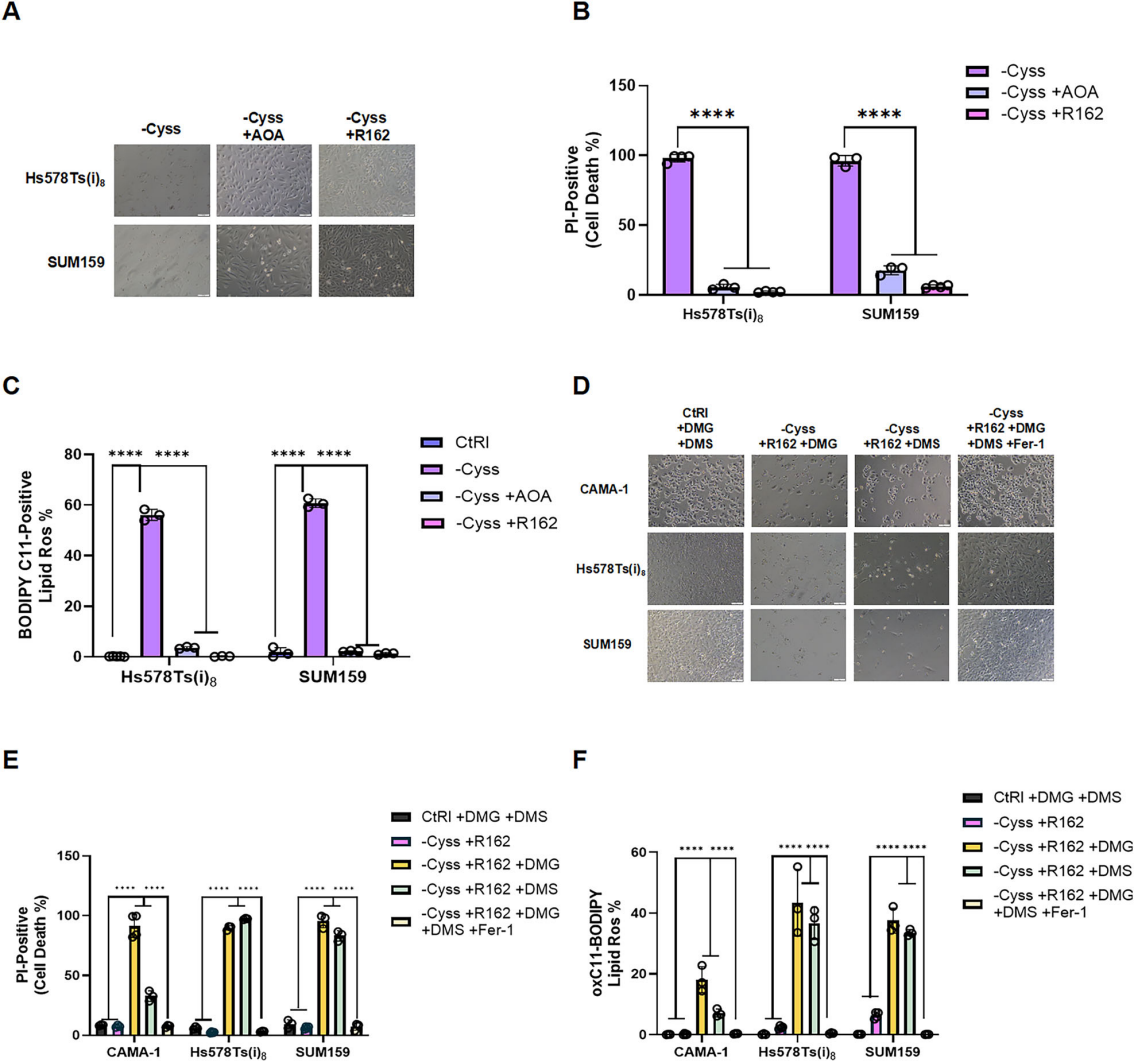
Glutamine anaplerosis regulates cystine deprivation-induced cell death by modulating lipid peroxidation. **A** Representative phase-contrast images of TNBC cells under cystine deprivation with GLUD1 inhibitor R162 (10 µM) or transaminase inhibitor AOA (50 µM) (scale bar = 100 µm). **B** Cell death assessed by the proportion of PI-positive cells and **C** lipid peroxidation levels measured by the percentage of oxidized BODIPY-C11-positive cells under the same treatments as in (**A**) (*n* = 3–4). **D** Representative phase-contrast images of cells treated with TCA cycle intermediates DMG (6 mM) and DMS (12 mM) in the context of cystine deprivation and GLUD1 inhibition, as well as the effect of ferroptosis inhibitor Fer-1 (5 µM) under the same conditions (scale bar = 100 µm). **E** Quantification of cell death and **F** lipid peroxidation under the same treatment conditions (*n* = 3–5). Data represent mean ± SD. Statistical analyses were performed using two-way ANOVA followed by Šídák’s multiple comparisons test (**B**, **C**, **E**, **F**), or unpaired *t*-test (bar graph in **C**). ****p < 0.0001.

Since glutamine availability is essential for cystine deprivation-induced cell death, we next investigated whether this dependence is linked to glutamine contribution to the TCA cycle via anaplerosis. To test this, we employed two inhibitors, aminooxyacetic acid (AOA) and R162, which block the conversion of glutamate to α-KG. As expected, both inhibitors prevented cell death under cystine deprivation in TNBC cells by reducing lipid peroxidation (Fig. 5A–C). Consistent with this, the addition of DMG and DMS eliminated the rescue effect seen with GLUD1 inhibition in TNBC cells by inducing lipid peroxidation (Fig. 5D–F). Interestingly, the addition of DMG and DMS also induced cell death in CAMA-1 cells, despite cystine deprivation alone having no effect on cell viability in this luminal BC cell line (Fig. 5D–F). This suggests that increased sensitivity to cystine deprivation in TNBC cells may be attributed to elevated glutamine anaplerosis compared to luminal breast cancer cells.

## Discussion

Triple-negative breast cancer (TNBC) cells exhibit a distinct metabolic phenotype compared to luminal breast cancer cells in vitro, including heightened reliance on extracellular amino acids [6], altered glucose metabolism [16, 17], and dysregulated lipid metabolism [18, 19]. In this study, we confirmed the dependence of TNBC cells on exogenous glutamine and cystine, as deprivation of either markedly suppressed their proliferation, while little effect on luminal breast cancer cells. Mechanistically, TNBC’s dependence on glutamine is attributed to an elevated requirement for glutamine anaplerosis, whereas cystine deprivation induces lipid peroxidation and ferroptotic cell death.

The expression of the cystine/glutamate antiporter SLC7A11 is closely linked to glutaminolysis, given its role in the 1:1 exchange of extracellular cystine for intracellular glutamate. Once imported, cystine is rapidly reduced to cysteine, which together with glutamate serves as a precursor for GSH synthesis. Due to this functional role, SLC7A11 is considered a therapeutic target in cancer for disrupting redox homeostasis [8]. In addition to antioxidant regulation, SLC7A11-mediated cystine transport also affects cellular bioenergetics, though with contrasting outcomes: inhibition of xCT promotes mitochondrial metabolism in CD44v^high^ head and neck squamous cell carcinoma (HNSCC) [20], while reduced OCR is observed upon SLC7A11 inactivation in KRAS-mutant lung cancer [21]. Similarly, Alexander et al. [22] demonstrated that SLC7A11 expression and cystine availability are critical regulators of glutamine anaplerosis in lung cancer.

To explore the role of SLC7A11 and cystine availability in regulating glutamine metabolism in TNBC, we examined its expression at both mRNA and protein levels. Public databases showed SLC7A11 enrichment in TNBC cells, which we validated via western blot across our panel of breast cancer cell lines. This finding aligns with prior observations [7]. Although combined inhibition of SLC7A11 and GLS has been shown to induce apoptosis and ferroptosis more effectively than targeting either alone in TNBC cells [11], our data show a different outcome. Using Hs578Ts(i)_8_, the most glutamine-dependent TNBC cell line, we demonstrate that pharmacological inhibition or knockdown of SLC7A11, as well as cystine deprivation, can rescue cells from glutamine deprivation-induced cell death, consistent with previous observations in lung cancer [9]. Mechanistically, we observed restoration of intracellular glutamate and TCA cycle intermediates under dual glutamine and cystine deprivation, a rescue effect not seen in luminal breast cancer cells. Additionally, cystine deprivation reduced the usage of exogenous glutamine in the TCA cycle.

Given the essential role of glutamine anaplerosis in TNBC and the reduction of this flux under cystine deprivation, we hypothesized that cystine dependence may in part reflect its modulation of glutamine metabolism. Addition of α-ketoglutarate, which restores TCA cycle function under glutamine deprivation, unexpectedly accelerated cell death under cystine deprivation. Conversely, inhibition of glutamine anaplerosis rescued TNBC cells from cystine deprivation-induced death, consistent with previous findings in fibroblasts [12, 13]. Mechanistically, glutaminolysis inhibition reduced ROS and lipid peroxidation levels triggered by cystine deprivation. Furthermore, supplementation with TCA cycle intermediates α-KG and succinate reversed the rescue effect of anaplerosis inhibition and induced lipid peroxidation-driven cell death, even in CAMA-1 cells, which are otherwise resistant to cystine deprivation, suggesting that differential sensitivity between TNBC and luminal cells may stem from their distinct glutamine metabolic flux.

Collectively, our findings reveal a dynamic metabolic interplay in TNBC cells, highlighting the dual reliance on cystine and glutamine and the pivotal role of SLC7A11 overexpression in regulating this dependence. We demonstrate that cystine deprivation under glutamine-deficient conditions restores intracellular glutamate and supports TCA cycle activity. Simultaneously, this dual deprivation mitigates ROS accumulation and lipid peroxidation. These insights underscore the therapeutic potential of targeting glutamine anaplerosis and cystine metabolism in TNBC and support future strategies that exploit their interconnected metabolic vulnerabilities.

## Materials and methods

### Cell culture

TNBC cell lines Hs578T and Hs578Ts(i)_8_ were kindly provided by Prof. Susan McDonnell (University College Dublin), and MDA-MB-231, SUM159, and luminal breast cancer cell lines CAMA-1, T47D, and ZR-75-1 were kindly provided by Prof. Michael Duffy (University College Dublin). All cell lines were cultured in high-glucose DMEM (4500 mg/L; D5671, Sigma-Aldrich) supplemented with 10% fetal bovine serum (10270106, Gibco) and 2 mM l-glutamine (25030-024, Gibco). Hs578T and Hs578Ts(i)_8_ cells were additionally supplemented with 10 µg/mL human insulin (I9278, Sigma-Aldrich). Cells were maintained at 37 °C in a humidified incubator with 5% CO_2_, and were passaged at 70–90% confluency.

### Alamar blue assay

Cells were seeded at a 5 × 10^3^ cells per well in 96-well plates and allowed to attach overnight. The following day, cells were treated as indicated. After the specified treatment duration, 10% (v/v) Alamar Blue reagent (DAL1100, Invitrogen) was added directly to each well, and plates were incubated at 37 °C in a humidified atmosphere with 5% CO_2_ for 6 h. Fluorescence intensity was measured using a microplate reader at 560 nm excitation and 590 nm emission wavelengths.

### Seahorse XF assay

Cells were seeded at a density of 1.5 × 10^4^ cells per well in a Seahorse XFp miniplate (103022-100, Agilent) and allowed to attach overnight. The assay was performed the following day according to the manufacturer’s protocol. Data were normalized to total protein content, measured using the BCA Protein Assay Kit (10741395, Thermo Fisher Scientific), and expressed as OCR per µg of protein.

### Gas chromatography mass spectrometry analysis

Cells were seeded at a density of 1.75 × 10^6^ cells per well in six-well plates and allowed to attach overnight. For isotope labeling, cells were incubated for 24 h (unless otherwise specified) in media supplemented with 2 mM U-^13^C_5_-glutamine (605166, Sigma-Aldrich) in either standard culture medium or cystine-free medium (21013024, Gibco) supplemented with 200 µM methionine (HY-13694, MedChemExpress). Metabolite extraction was performed by rapid quenching using a cold methanol:water:chloroform (5:2:5, v/v/v) solution, with l-norvaline (0.1 µg; N7627, Sigma) added as an internal standard.

Dried polar metabolites were derivatized in 2% (w/v) methoxyamine hydrochloride (Thermo Scientific) in pyridine and incubated at 37 °C for 60 min. Samples were then silylated with N-tertbutyldimethylsilyl-N-methyltrifluoroacetamide (MTBSTFA) with 1% tert-butyldimethylchlorosilane (tBDMCS) (Regis Technologies) at 45 °C for 30 min. Polar derivatives were analyzed by GC–MS using a DB-35MS column (30 m × 0.25 mm i.d. × 0.25 μm, Agilent J&W Scientific) installed in an Agilent 7890B gas chromatograph (GC) interfaced with an Agilent 5977 A mass spectrometer (MS) with an XTR ion source using the following temperature program: 100 °C initial, increase by 5 °C/min to 215 °C, increase by 10 °C/min to 320 °C and hold for 4 min. The percent isotopologue distribution of each polar metabolite was determined and corrected for natural abundance via in-house MATLAB scripts using algorithms adapted from Fernandez et al. [23] and mass fragments outlined in Cordes et al. [24].

### siRNA transfection

Cells were seeded at a density of 2 × 10^6^ cells per well in six-well plates using serum-free and penicillin–streptomycin-free (SF/PSF) DMEM and allowed to attach overnight before transfection. For gene knockdown, cells were transfected with Silencer Select siRNA targeting SLC7A11 (4390824, Assay ID: s24291, Thermo Fisher Scientific) or Silencer Select Negative Control siRNA (4427038, Thermo Fisher Scientific). Transfection complexes were prepared in two separate tubes, each containing 1 mL SF/PSF DMEM, mixed with Lipofectamine RNAiMAX transfection reagent (5 µL/mL; 13778030, Thermo Fisher Scientific) and siRNA (25 nM). After 15 min of incubation at room temperature on a roller, the complexes were added to the cells. Cells were incubated for 24 h prior to the indicated downstream treatments.

### Flow cytometry analysis

Cells were seeded at a density of 1 × 10^5^ cells per well in 12-well plates and allowed to attach overnight. Following the indicated treatments, intracellular ROS and lipid peroxidation were assessed using H_2_DCFDA (10 µM; D6883, Sigma-Aldrich) and BODIPY-C11 (2 µM; D3861, Invitrogen), respectively. Dyes were added 30 minutes prior to the end of treatment, followed by immediate sample collection. To assess cell viability after per treatment, cells were washed twice with PBS and scraped into fresh PBS for collection. Propidium iodide (PI) staining (2 µg/mL; P3566, Invitrogen) was performed post-sampe collection to assess membrane integrity and identify dead cells. Samples were analyzed by flow cytometry using a BD FACS Canto or Accuri C6 cytometer.

### Western blot analysis

Cells were washed twice with cold PBS and lysed in RIPA buffer (R0278, Sigma-Aldrich), followed by vortexing and incubation on ice for 30 min. Lysates were centrifuged at 14,000 rpm for 10 min at 4 °C to remove debris. Protein concentration was determined using the BCA Protein Assay Kit (Thermo Fisher Scientific). Equal amounts of protein were separated on 8–12% SDS–PAGE gels and transferred onto PVDF membranes (88518, Thermo Fisher Scientific). Membranes were blocked and incubated with primary antibodies diluted in 5% nonfat dry milk in Tris-buffered saline with 0.05% Tween-20 (TBST) at 4 °C overnight. After washing, membranes were incubated with HRP-conjugated secondary antibodies (Mouse: W4021; Rabbit: W4022; Promega) and visualized using ECL detection reagent (34096, Thermo Fisher Scientific).

### Antibodies and Inhibitors

CB839 (HY-12248), Erastin (HY-15763), Fer-1 (HY-100579), Sulfasalazine (HY-14655), Trolox (HY-101445), R162 (HY-103096), and AOA (HY-107994) were purchased from MedChemExpress. Oligomycin, FCCP, Rotenone, and antimycin A (103010-100) are contained in the Cell Mito Stress Test Kit, Agilent. Dimethyl ɑ-KG (349631) and dimethyl succinate (8201500250) were purchased from Sigma-Aldrich.

Antibodies GLS1 (88964), GLUD1/2 (12793), PINK1 (6946), and β-Actin (4967) were purchased from Cell Signalling; SLC7A11 (26864-1-AP) was purchased from ProteinTech; and TOMM20 (ST1705) was purchased from Sigma-Aldrich.

### Statistical analysis

Details of all statistical analyses performed can be found in the figure legends. Data were expressed as mean ± SD. Significance was defined as follows: *p < 0.05, **p < 0.005, ***p < 0.0005, ****p < 0.0001. All depicted data points are biological replicates taken from distinct samples. Each figure consists of a minimum of three independent experiments from multiple biological replicates.

## Supplementary information

Supplementary figure legends

Figure S1.1

Figure S1.2

Figure S2

Figure S3

Figure S4

Figure S5

## Data Availability

All data generated and analyzed during this study are included in this article and its supplementary information files. Further details or raw data are available from the corresponding author upon reasonable request.
