# Supplementary material for: Metabolic interplay between exogenous cystine and glutamine dependence in triple-negative breast cancer
Source: Cell Death Discov. 2025 Oct 6;11:430. doi: 10.1038/s41420-025-02714-3 (PMC12501009; doi:10.1038/s41420-025-02714-3)
Supplement: Supplementary file 1 — Supplementary figure legends [file 41420_2025_2714_MOESM1_ESM.pdf]

Figure S1.1. Comparison of migration and invasion potential between Hs578T and Hs578Ts(i)$_8$ cells and the effect of CB839 on invasion. (A) Cell migration assessed by scratch assay over a time course; percentage migration was calculated as the remaining cell-free area relative to the initial scratch area at 0 h. (B) Transwell migration assay quantifying migrated cells after 16 hours, stained with crystal violet. (C) Cell invasion assessed by transwell invasion assay; quantification was based on absorbance of crystal violet stain dissolved in 10% acetic acid after 22 hours. (D) Effect of CB839 on invasion potential, assessed by transwell assay and normalized to vehicle control (untreated) cells. Data represent mean ± SD (n=3). Statistical analyses were performed using two-way ANOVA with Šídák's multiple comparisons test (A), or unpaired t-test (B-D). ***$p < 0.001$, **$p < 0.01$, *$p < 0.05$.

Figure S1.2 Assessment of GLS1 expression in breast cancer cells and response to cystine deprivation. (A) Relative GLS1 RNA expression levels across breast cancer cell lines using the CCLE database, normalized to the median expression. (B) GLS1 expression from the HMS LINCS public dataset. (C) Protein levels of GLS1 across the panel of indicated breast cancer cell lines. (D) Effect of ferroptosis inhibitor Fer-1 (5 µM) on TNBC cells under cystine-deprived conditions (n=3-5). Data represent mean ± SD.

Figure S2. OCR profiles of a panel of luminal and TNBC cell lines. Real-time OCR was measured following sequential injections of glutamine (2 mM), oligomycin (1.5 µM), FCCP (2 µM for Hs578Ts(i)$_8$ and SUM159; 0.5 µM for the remaining cell lines), and a combination of antimycin A (0.5 µM) and rotenone (0.5 µM). OCR was normalized to protein content and expressed as pmol/min/µg protein in (A) TNBC cell lines and (B) luminal breast cancer cell lines. Data represent mean ± SD (n=3).

Figure S3. Expression of SLC7A11 in breast cancer cells and metabolic response to nutrient deprivation. (A) Relative RNA expression levels of SLC7A11 normalized to the median across all cell lines using the CCLE database. (B) RNA expression data from the HMS LINCS public database. (C) Protein expression of SLC7A11 in the panel of indicated breast cancer cell lines. (D) Knockdown efficiency of SLC7A11 in Hs578Ts(i)$_8$ cells. (E) Normalized ion counts of TCA cycle intermediates in CAMA-1 cells under glutamine deprivation and combined glutamine and cystine deprivation conditions, data represent mean ± SD from at least three independent experiments.

Figure S4. Mitophagy induced by FCCP and glutamine deprivation rescues TNBC cells from cystine deprivation by reducing lipid peroxides. (A) Representative phase-contrast images of Hs578Ts(i)$_8$ cells treated with

FCCP (2 μM) or glutamine deprivation under cystine-deprived conditions. (B) Quantification of cell death by PI-positive cells after 48 hours of treatment. (C) Lipid peroxidation levels assessed by the percentage of oxidized BODIPY-C11-positive cells after 16 hours. (D) Western blot analysis of mitophagy marker PINK1 and mitochondrial membrane marker TOMM20 under the same treatment conditions. Data represent mean ± SD (n=3). Statistical analyses were performed using one-way ANOVA with Tukey's multiple comparisons test (B,C). ****$p < 0.0001$.

Figure S5. Original western blots. Original western blots of (A) GLS1 and (B) SLC7A11 among different breast cancer cell lines. (C) GLUD under indicated conditions and cell lines. (D) PINK1 and (B) TOMM20 under indicated conditions in Hs578Ts(i)$_8$ cells.
