# Supplementary figures and images for: Metabolic interplay between exogenous cystine and glutamine dependence in triple-negative breast cancer
Source: Cell Death Discov. 2025 Oct 6;11:430. doi: 10.1038/s41420-025-02714-3 (PMC12501009; doi:10.1038/s41420-025-02714-3)

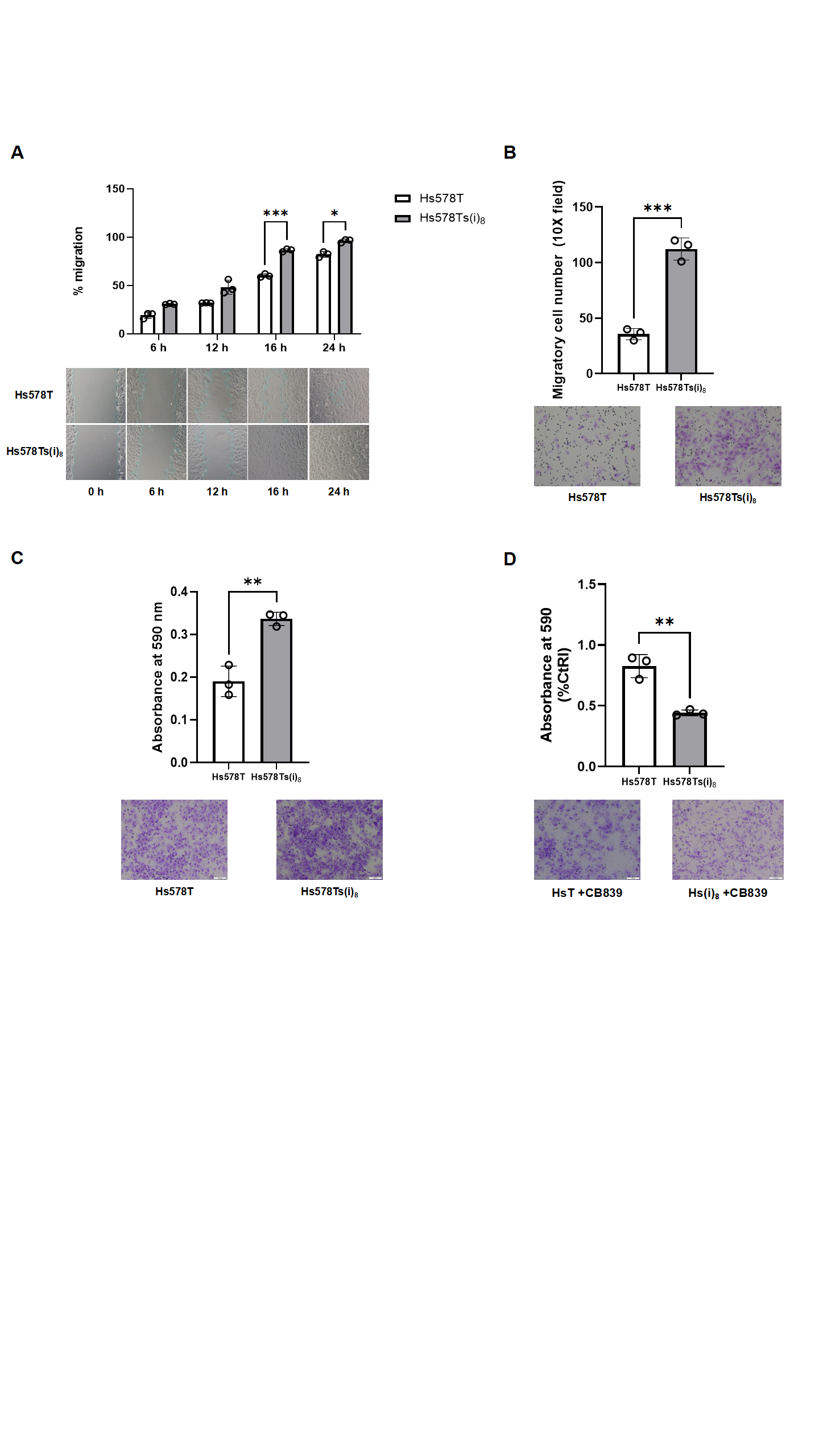

Supplement: Supplementary file 2 — Figure S1.1 [file 41420_2025_2714_MOESM2_ESM.png]

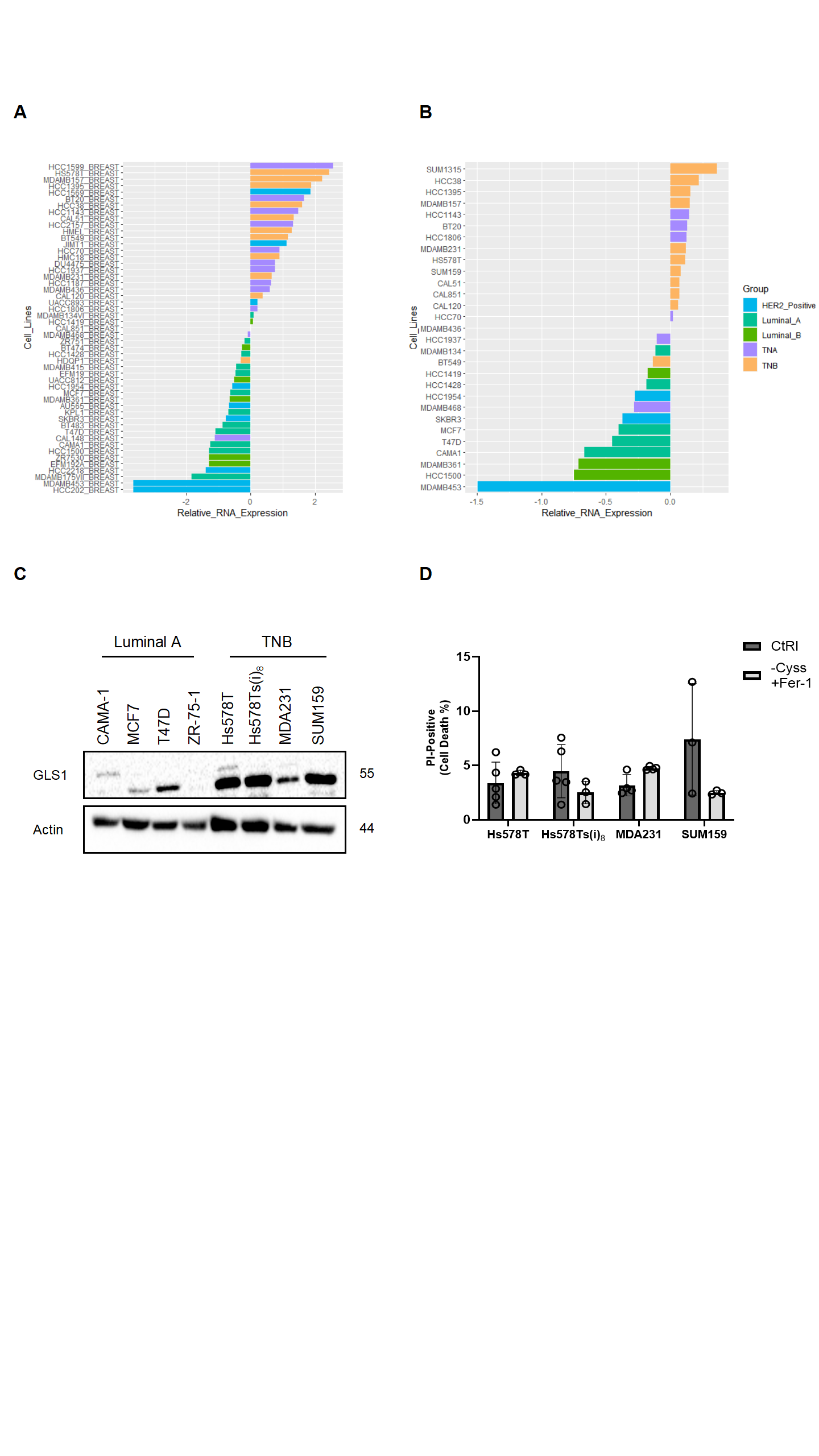

Supplement: Supplementary file 3 — Figure S1.2 [file 41420_2025_2714_MOESM3_ESM.png]

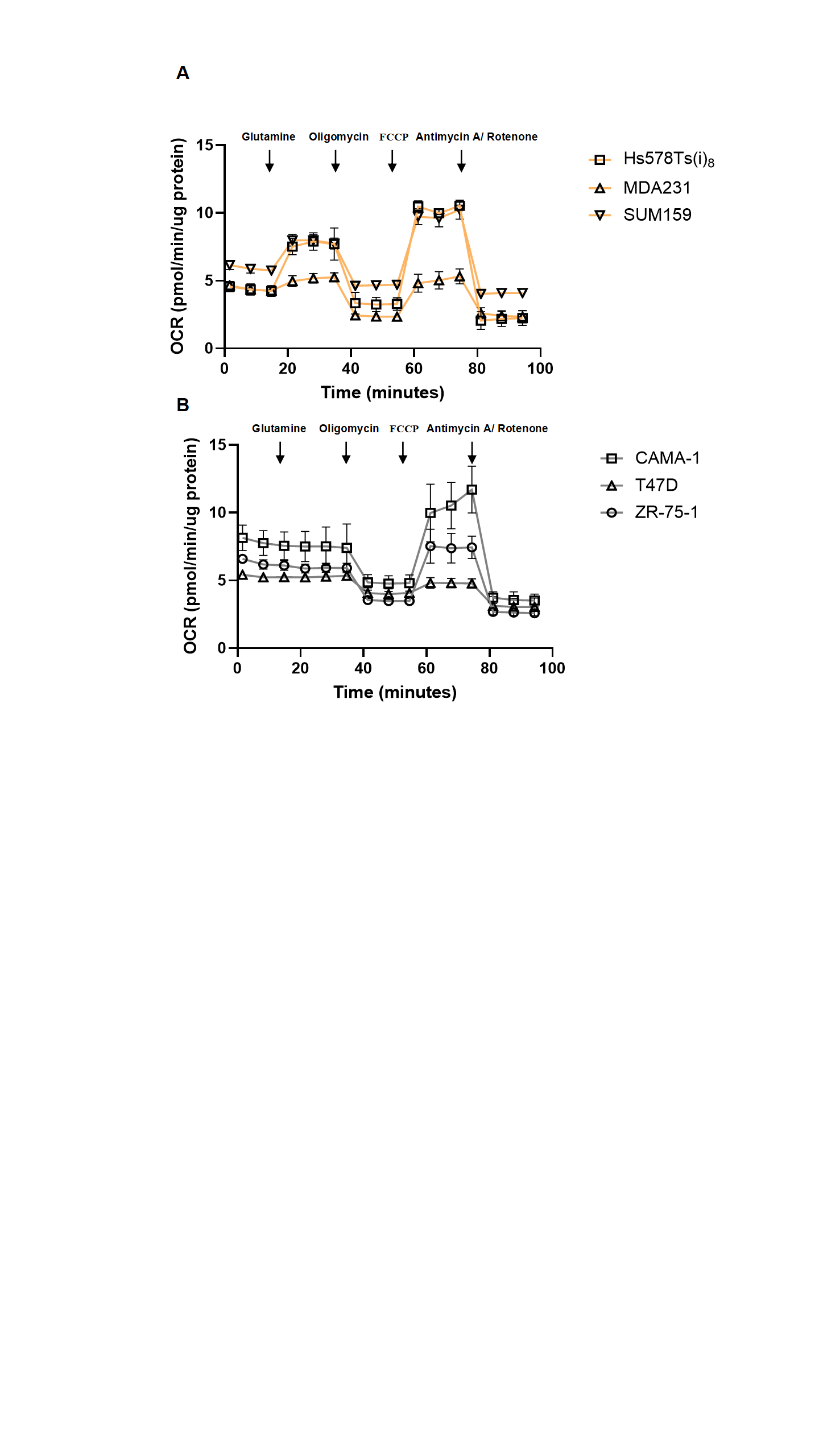

Supplement: Supplementary file 4 — Figure S2 [file 41420_2025_2714_MOESM4_ESM.png]

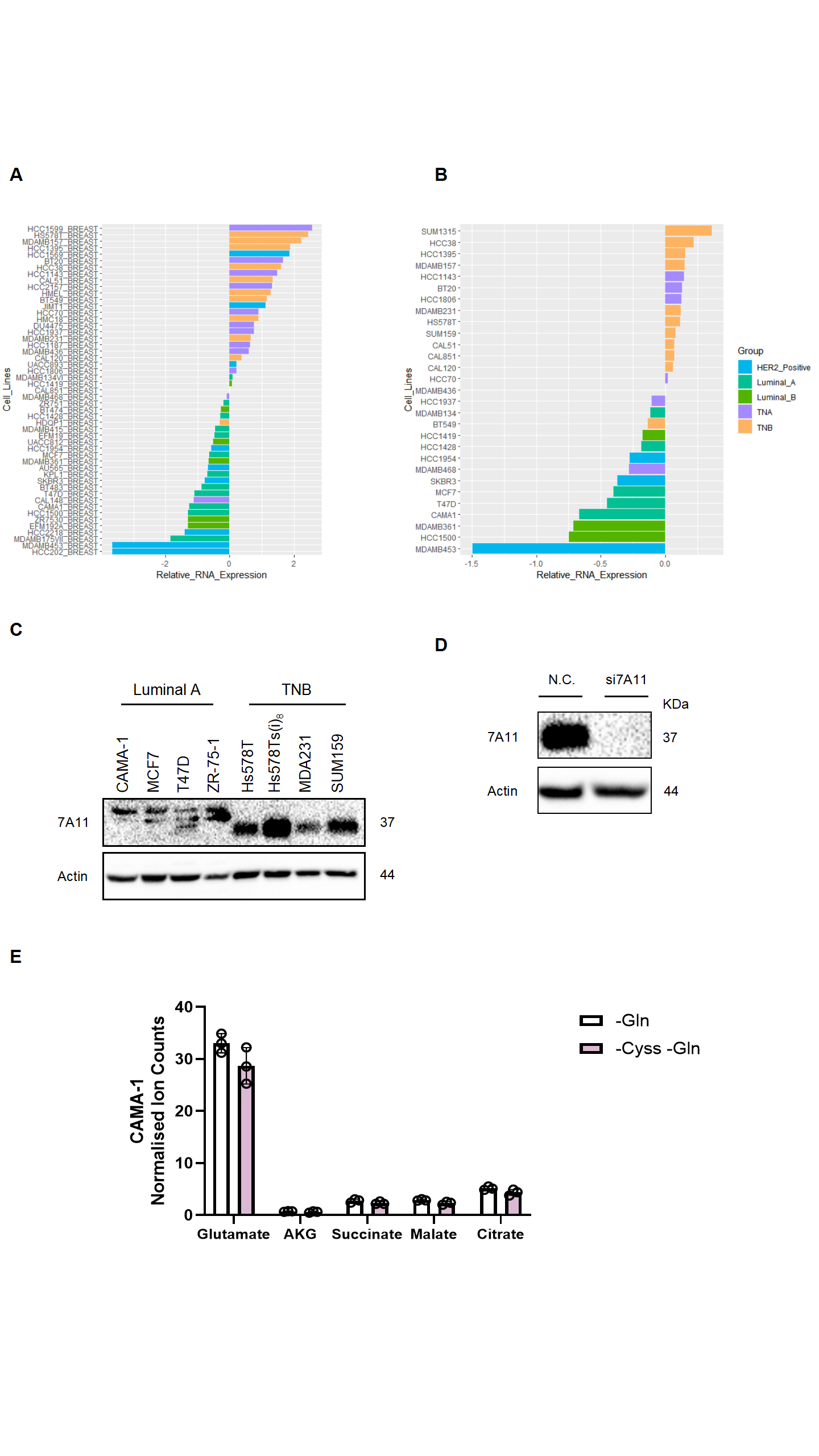

Supplement: Supplementary file 5 — Figure S3 [file 41420_2025_2714_MOESM5_ESM.png]

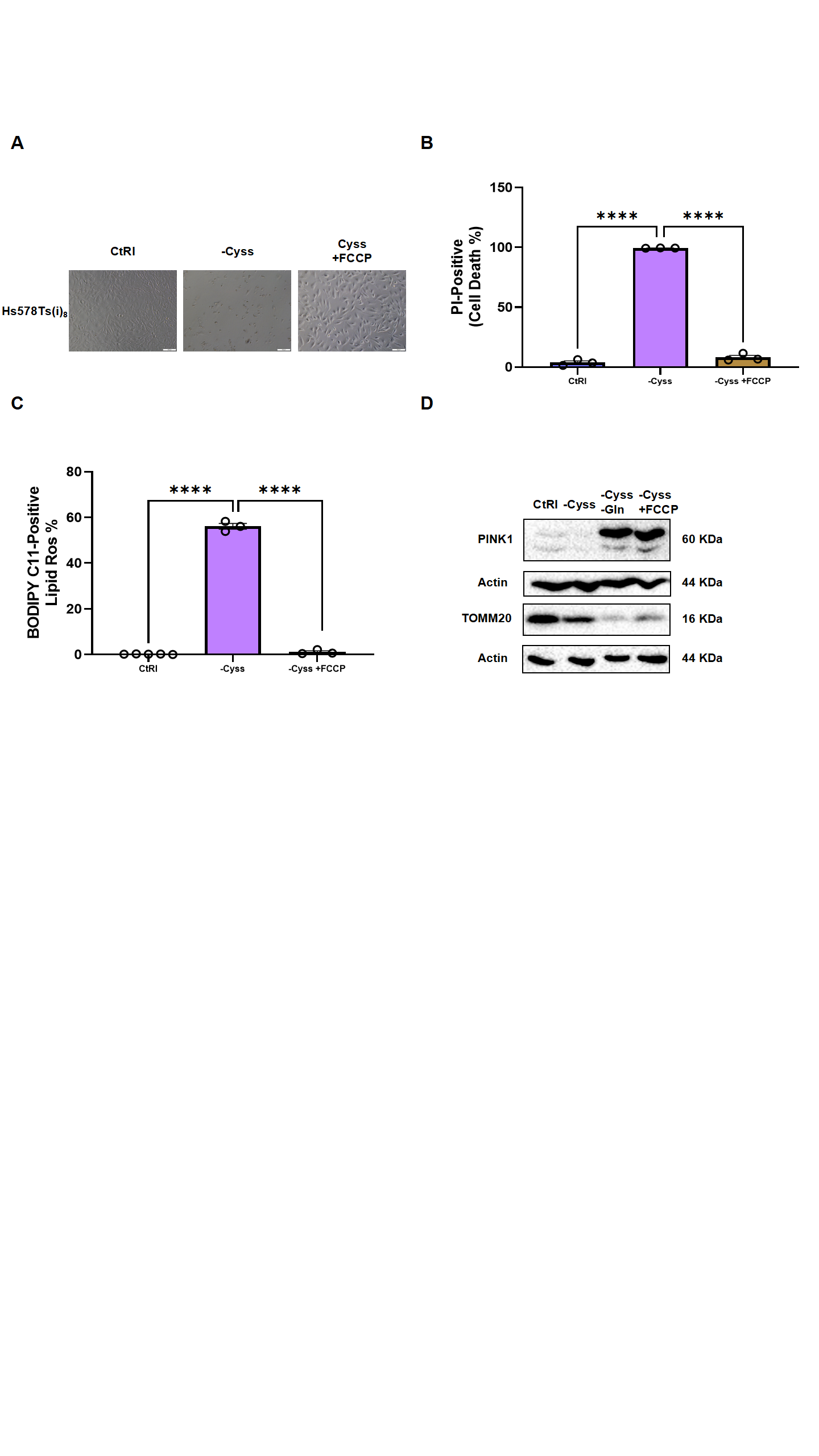

Supplement: Supplementary file 6 — Figure S4 [file 41420_2025_2714_MOESM6_ESM.png]

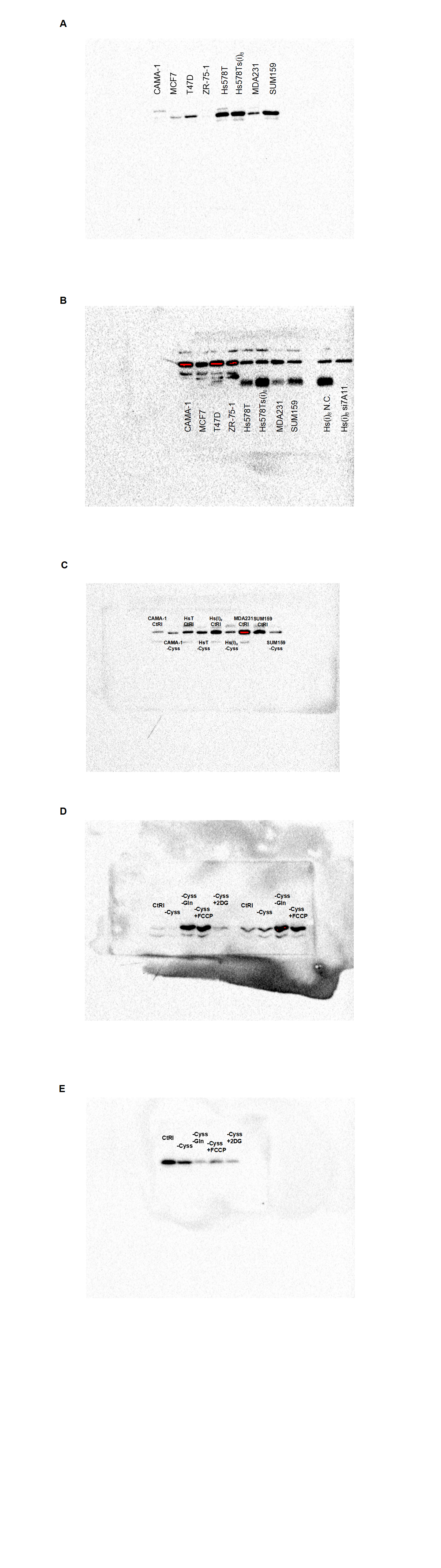

Supplement: Supplementary file 7 — Figure S5 [file 41420_2025_2714_MOESM7_ESM.png]
